# Insights into adherence to medication and lifestyle recommendations in an international cohort of patients with catecholaminergic polymorphic ventricular tachycardia

**DOI:** 10.1093/europace/euae044

**Published:** 2024-02-13

**Authors:** Puck J Peltenburg, Lieke M van den Heuvel, Dania Kallas, Cheyanne Bell, Isabelle Denjoy, Elijah R Behr, Ella Field, Janneke A E Kammeraad, Sing-Chien Yap, Vincent Probst, Michael J Ackerman, Nico A Blom, Arthur A M Wilde, Sally-Ann B Clur, Christian van der Werf

**Affiliations:** Deparment of Clinical and Exprimental Cardiology, Heart Center, Amsterdam UMC, University of Amsterdam, Amsterdam, The Netherlands; Department of Pediatric Cardiology, Emma Children’s Hospital, Amsterdam UMC, University of Amsterdam, Amsterdam, The Netherlands; Department of Genetics, University Medical Centre Utrecht, Utrecht, The Netherlands; Netherlands Heart Institute, Utrecht, the Netherlands; Department of Pediatrics, BC Children’s Hospital, University of British Columbia, Vancouver, Canada; Windland Smith Rice Sudden Death Genomics Laboratory, Division of Heart Rhythm Services and Pediatric Cardiology, Departments of Cardiovascular Medicine, Pediatric and Adolescent Medicine, and Molecular Pharmacology & Experimental Therapeutics, Mayo Clinic, Rochester, Minnesota, USA; Service de Cardiologie et CNMR Maladies Cardiaques Héréditaires Rares, Hôpital Bichat APHP, Université de Paris, Paris, France; Cardiovascular Clinical Academic Group and Cardiology Research Centre, Molecular and Clinical Sciences Research Institute, St. George’s, University of London, St. George’s University Hospitals NHS Foundation Trust, Cranmer Terrace, London, UK; Department of Pediatric Cardiology, Great Ormond Street Hospital, London, UK; Department of Pediatric Cardiology, Erasmus MC - Sophia, Rotterdam, The Netherlands; Department of Cardiology, Cardiovascular Institute, Erasmus MC, Rotterdam, The Netherlands; Service de cardiologie, Université de Nantes, CNRS, INSERM, l’institut du thorax, Nantes, France; Windland Smith Rice Sudden Death Genomics Laboratory, Division of Heart Rhythm Services and Pediatric Cardiology, Departments of Cardiovascular Medicine, Pediatric and Adolescent Medicine, and Molecular Pharmacology & Experimental Therapeutics, Mayo Clinic, Rochester, Minnesota, USA; Department of Pediatric Cardiology, Emma Children’s Hospital, Amsterdam UMC, University of Amsterdam, Amsterdam, The Netherlands; Department of Pediatric Cardiology, Willem-Alexander Children’s Hospital, Leiden University Medical Centre, Leiden, The Netherlands; Deparment of Clinical and Exprimental Cardiology, Heart Center, Amsterdam UMC, University of Amsterdam, Amsterdam, The Netherlands; Department of Pediatric Cardiology, Emma Children’s Hospital, Amsterdam UMC, University of Amsterdam, Amsterdam, The Netherlands; Deparment of Clinical and Exprimental Cardiology, Heart Center, Amsterdam UMC, University of Amsterdam, Amsterdam, The Netherlands

**Keywords:** Catecholaminergic polymorphic ventricular tachycardia, Adherence

## Abstract

**Aims:**

In patients with catecholaminergic polymorphic ventricular tachycardia (CPVT), a rare inherited arrhythmia syndrome, arrhythmic events can be prevented by medication and lifestyle recommendations. In patients who experience breakthrough arrhythmic events, non-adherence plays an essential role. We aimed to investigate the incidence and potential reasons for non-adherence to medication and lifestyle recommendations in a large, international cohort of patients with CPVT.

**Methods and results:**

An online multilingual survey was shared with CPVT patients worldwide by their cardiologists, through peer-recruitment, and on social media from November 2022 until July 2023. Self-reported non-adherence was measured using the validated Medication Adherence Rating Scale (MARS) and a newly developed questionnaire about lifestyle. Additionally, validated questionnaires were used to assess potential reasons for medication non-adherence. Two-hundred-and-eighteen patients completed the survey, of whom 200 (92%) were prescribed medication [122 (61%) female; median age 33.5 years (interquartile range: 22–50)]. One-hundred-and-three (52%) were prescribed beta-blocker and flecainide, 85 (43%) beta-blocker, and 11 (6%) flecainide. Thirty-four (17%) patients experienced a syncope, aborted cardiac arrest or appropriate implantable cardioverter defibrillator shock after diagnosis. Nineteen (13.4%) patients were exercising more than recommended. Thirty (15%) patients were non-adherent to medication. Female sex [odds ratio (OR) 3.7, 95% confidence interval (CI) 1.3–12.0, *P* = 0.019], flecainide monotherapy compared to combination therapy (OR 6.8, 95% CI 1.6–31.0, *P* = 0.010), and a higher agreement with statements regarding concerns about CPVT medication (OR 1.2, 95% CI 1.1–1.3, *P* < 0.001) were independently associated with non-adherence.

**Conclusion:**

The significant rate of non-adherence associated with concerns regarding CPVT-related medication, emphasizes the potential for improving therapy adherence by targeted patient education.

## Graphical Abstract

**Graphical Abstract:**
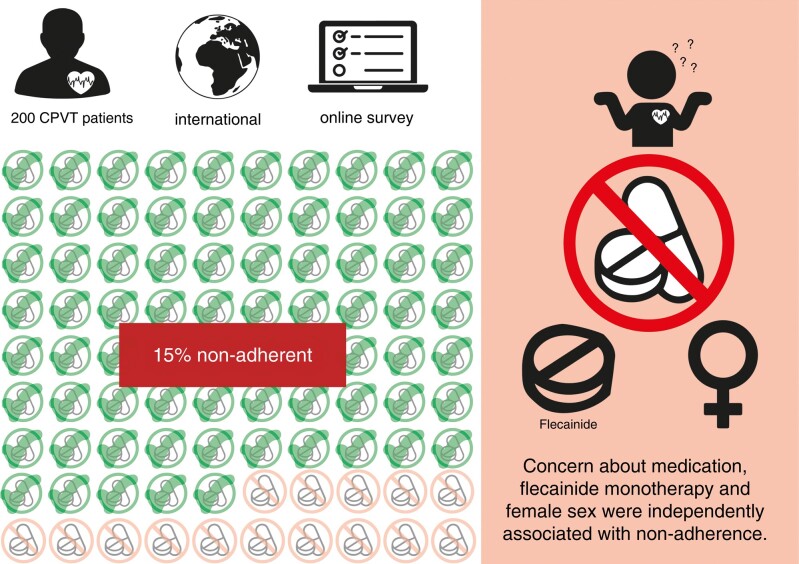

**What’s new?**

- Around 15% of a representative international cohort of catecholaminergic polymorphic ventricular tachycardia (CPVT) patients is non-adherent to their CPVT medication.
- Concern about CPVT medication is independently associated with non-adherence and, therefore, addressing these concerns might serve as a ground to improve patient education and reduce non-adherence.

## Introduction

Catecholaminergic polymorphic ventricular tachycardia (CPVT) is a rare inherited arrhythmia syndrome, in which potentially life-threatening arrhythmic events are often triggered by adrenergic situations, such as exercise or emotional stress.^1^ It is well established that medication, specifically non-selective beta-blockers, such as nadolol and propranolol, and flecainide combined with lifestyle recommendations (in particular sports restrictions in high-risk patients) are effective in reducing the risk for arrhythmic events.^2–5^ As such, these are the mainstay in the CPVT treatment.^6^ However, a significant number of patients still experience breakthrough arrhythmic events,^7–10^ prompting a next step in the treatment escalation, i.e. a left cardiac sympathetic denervation and/or implantation of an implantable cardioverter defibrillator.^6^ These arrhythmic events are frequently associated with medication non-adherence,^2,8,11,12^ potentially combined with non-adherence to lifestyle recommendations. It is thus essential to understand the incidence and risk factors for therapy non-adherence in the CPVT population. These insights may ultimately result in interventions to reduce therapy non-adherence and subsequent risk for arrhythmic events.

Previously, a survey study in patients with an inherited cardiac disease, including 5 (4%) patients with CPVT, showed that a younger age and an underlying inherited arrhythmia syndrome were associated with non-adherence to beta-blockers.^13^ Adherence was worse in those patients who had high concerns and low necessity beliefs about their beta-blocker.^13^ In a large cohort of congenital long QT syndrome patients, an inherited arrhythmia syndrome in which patients are also at risk for lethal arrhythmic events and beta-blockers are the cornerstone of treatment, reduced medication adherence was noted in more than a third of the patients.^14^ These studies underline the importance of therapy non-adherence in patients with inherited arrhythmia syndromes, including CPVT.

Additionally, patients with CPVT are usually advised to refrain from competitive sports, strenuous exercise, and exposure to stressful environments.^6^ This advice is mainly based on expert opinion, but it has been suggested that the risk for arrhythmic events during sports participation in well-treated and well-informed patients is acceptable.^15^ Accordingly, other guidelines tend to be more lenient.^16^ This emphasizes that sports recommendation given by cardiologists may differ worldwide, and, in addition, patients’ adherence to these recommendations is currently unknown.

With this international online survey study, we aimed to study the incidence and risk factors associated with self-reported non-adherence to medical therapy and lifestyle recommendations within a large cohort of patients with CPVT.

## Methods

### Study design and consent

This prospective anonymous survey study was available online from November 2022 until July 2023 in Dutch, English, and French. The survey was hosted on an electronic data capture system, Castor.^17^ Patients diagnosed with CPVT aged 12 years or older were recruited to enter the survey. Informed consent of the participants and of the participants’ parents for children between 12 and 16 years of age was obtained. Official institutional review of this study was waived by the Medical Ethics Review Committee of the Academic Medical Center, Amsterdam, the Netherlands.

### Questionnaires and primary outcomes

The survey consisted of five sections. The first three sections were mandatory, the fourth and fifth were optional.

1. General CPVT-related questions
  This included questions regarding the CPVT diagnosis, therapy, symptoms, and demographic characteristics. (See Supplementary Methods for a detailed overview of CPVT related questions.)
2. Adherence to medical therapy
  Adherence was measured using a validated questionnaire that was previously used in the field of inherited cardiac diseases, the Medication Adherence Report Scale-5 (MARS-5).^13^ The MARS-5 is designed to reveal self-reported use of medication and it contains five 5-point Likert scale questions related to medication intake. The first question reflects unintentional non-adherence and the subsequent four questions reflect intentional non-adherence. The MARS-5 showed good internal consistency in adults with various conditions.^18^ The internal consistency of the MARS-5 in children with asthma was low,^19^ but currently no other validated alternatives are available for children. The total score of all MARS-5 5-point Likert scale questions combined was dichotomized to differentiate adherent (total score ≥23) from non-adherent (total score <23) participants. This cut-off was previously defined and is used in several studies, including studies of patients using cardiovascular medications and of children.^20,21^
3. Thoughts and beliefs about medication and CPVT.
  Two questionnaires were used to assess patients’ thoughts and beliefs regarding medication and CPVT: the Beliefs about Medications Questionnaire (BMQ)^22,23^ and the Brief Illness Perception Questionnaire (BIPQ).^24,25^ The BMQ contained a CPVT-specific medication section and a section about medication in general. Four different subdomains of questions within the BMQ can be distinguished: concern about specific medication, necessity of specific medication, overuse of medication in general, harm of medication in general.^23^ The BIPQ contained seven questions about the participants’ CPVT experiences, that could be rated from 0 (no effect) to 10 (severe effect).
4. Lifestyle
  A self-constructed questionnaire regarding lifestyle was included. This survey contained questions about the lifestyle advices that the participants received from their physician, their actual lifestyle, and the feelings about their lifestyle. (See Supplementary Methods for complete list of the questions.)
5. Two self-constructed optional free-text questions asking the participants about their ideas on improvements for the care for people with CPVT and on their opinion of the survey. Participants’ quotations from these questions are shown to illustrate the results.

The original survey was drafted in Dutch. It was translated to English by one author (P.J.P.) and revised by a native English speaker (S.A.B.C.). The survey was translated from Dutch to French by two official medical translators. When available, the official versions of the MARS-5, BMQ, and BIPQ questionnaires were used.

### Study pilot

A pilot version of this survey was completed by four CPVT patients (three patients aged >16 years, and one patient 12–16 years old who completed the survey with a parent). With all four participants a phone interview was conducted to discuss the survey content. Based on the feedback gained, improvements in the wording were made to the final version of the survey. In the survey for children from 12 to 16 years old, the BMQ-general questionnaire was deemed inappropriate for their age and was therefore discarded in the final version.

### Recruitment

As this was an open online survey study, participants were recruited using three different methods. Firstly, treating physicians from six tertiary referral centres worldwide promoted the survey in their outpatients clinic and/or sent an information email about the survey to their CPVT patients. Secondly, the survey was promoted on social media by CPVT-specific social media platforms, channelling only patients with CPVT (for details, see Supplementary Methods). Lastly, after completing the survey, the flyer and the links to the surveys were shared with participants requesting them to promote the survey to their family members or other acquaintances diagnosed with CPVT.

### Study cohort

We aimed to include at least 213 CPVT patients to complete the survey. This sample size goal was based on previous studies using a similar recruitment method, the estimated prevalence of CPVT, and the number of patients in the International CPVT Registry.^5^ A detailed breakdown of this estimated sample size calculation can be found in the Supplementary Methods.

### Response validation

The survey was open to everyone with the link and responses were collected anonymously. To reduce the risk of sampling bias (i.e. the survey being entered by a participant who does not have CPVT), we focused on specific CPVT populations when sharing the survey on social media. Furthermore, multiple checks were built into the survey verifying the CPVT diagnosis and asking participants to close the survey if they did not have CPVT. Dependencies were integrated into the survey to ensure the accuracy of critical responses to important questions (e.g. concerning the use of CPVT medication, if a participant selected ‘no’ to this question, Section 2 of the survey was not shown and instead a message prompted: ‘If you don't use any CPVT medicine, please click the button ‘Next’ at the bottom of this page. If you do use CPVT medicine, click the button ‘Previous’ to correct your answer to question 16 of Part 1 (Do you use medication for CPVT?).’). Because patients could have potentially received an invitation through multiple channels and therefore, accessed the survey twice, all survey entries with the same sex, current age, age at diagnosis, treating hospital, and age at first start of medication were manually checked for duplicate entries. The most recent duplicate entry was excluded. Lastly, we assessed whether the events reported by participants followed a feasible chronological pattern.

### Statistical analysis

Categorical variables are described as numbers and percentages. The categorical variables of the lifestyle section are described as numbers/total number of received answers and percentages based on the total number of received answers. Continuous variables are described as mean ± standard deviation (SD) or median [interquartile range (IQR)], as appropriate. Categorical variables in two groups were compared using a χ^2^ test or Fisher’s exact test, as appropriate, and Bonferroni correction was applied when necessary. Ordinal variables were compared between two groups using a χ^2^ test. Continuous variables between two groups were compared using a Student’s *t*-test or Mann–Whitney *U* test, as appropriate. Logistic regression was performed comparing non-adherent participants vs. adherent participants with demographics, and thoughts and beliefs about CPVT and medications as independent variables. Variables that differed between the two groups on a significance level with *P* < 0.10 were assessed in a multivariable model to correct for confounders. R version 4.2.1 (R Project for Statistical Computing, Vienna, Austria) was used for the statistical analyses. The following R packages were used for the creation of the figures: ‘ggaluvial’ and ‘ggplot’.

## Results

### Study population

**Figure 1:**
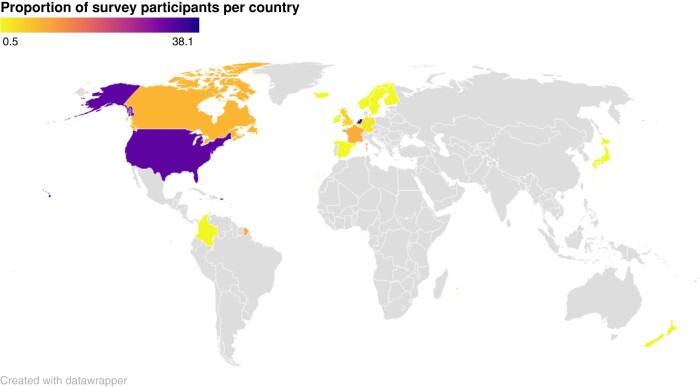
Proportion of survey participants per country. This figure represents the residing country of the survey participants in percentage of the total survey population. The majority of the survey participants were from the Netherlands (*n* = 85 [38.5%] or from the USA (*n* = 73 [33.0%]).

A total of 222 surveys were completed. Four survey entries were excluded: one survey participant was the parent of a child that was deceased, and three survey entries were identified as duplicate entries from the same participant and were excluded. All remaining survey entries (*n* = 218) followed a feasible chronological order of events. Most participants (*n* = 121, 55.5%) were referred to the survey by their treating physician, followed by 38 (17.4%) participants through social media, 34 (15.6%) through a newsletter, and 24 (11.0%) were referred to the survey by an acquaintance. Patients worldwide responded to the survey, and the majority of the patients were from either the Netherlands [*n* = 83 (38.1%)] or the USA [*n* = 71 (32.6%), *Figure 1*]. The median age of the survey participants was 34 [22.0–51.0] years, 133 (61.0%) participants were female, and 78 (35.8%) participants were initially evaluated as part of family screening, while 103 (47.2%) were initially evaluated due to cardiac symptoms (*Table 1*).

**Table 1:** Demographics and CPVT characteristics of all survey respondents

	Complete cohort (*n* = 218)
Current age (median [IQR])	34.0 [22.0–51.0]
Gender, female (%)	133 (61.0)
Genotype (%)	
Unknown	38 (17.4)
Genotype-negative	12 (5.5)
Genotype-positive	168 (77.1)
*RYR2* variant carrier (%)	116 (69.0)
Age at diagnosis (median [IQR])	25.0 [13.0–40.0]
Autism (%)	14 (7.2)
Reason of presentation (%)	
CPVT cascade screening	78 (35.8)
Incidental finding	20 (9.2)
Screening because of sudden cardiac death in family	17 (7.8)
Cardiac symptoms	103 (47.2)
Worst symptom prior to diagnosis (%)	
Aborted cardiac arrest	36 (16.5)
Asymptomatic	82 (37.6)
Palpitations	27 (12.4)
Syncope	73 (33.5)
Symptoms after diagnosis^a^ (%)	37 (17.0)
Near-fatal symptoms after diagnosis^b^ (%)	24 (11.0)
ICD implanted (%)	49 (22.5)
Left cardiac sympathetic denervation (%)	29 (13.3)
Follow-up frequency (%)	
Never	13 (6.0)
Once per 2 or 3 years	34 (15.6)
Once a year	109 (50.0)
More than once a year	49 (22.5)
Other	13 (6.0)

^a^Defined as syncope, aborted cardiac arrest, or appropriate implantable cardioverter defibrillator shock.

^b^Defined as aborted cardiac arrest or appropriate implantable cardioverter defibrillator shock.

CPVT, catecholaminergic polymorphic ventricular tachycardia; ICD, implantable cardioverter defibrillator; IQR, interquartile range.

Eighteen (8.3%) participants did not use CPVT medication. Of these, 10 (55.6%) were intentionally not prescribed medication by their (paediatric) cardiologist and 6 (33.3%) refused to take medication or stopped medication due to side effects.

### Participants using medication

A total of 200 participants (median age 33.5 [22.0–50.0] years) used medication for CPVT, of whom 122 (61.0%) were female and 109 (54.5%) reported to be a carrier of a *RYR2* gene variant. Most participants (*n* = 103, 47.2%) first visited the cardiologist due to symptoms; 32 (17.0%) experienced a sudden cardiac arrest, and 62 (31.0%) had a syncopal event prior to the diagnosis. Forty-six (23.0%) participants had an implantable cardioverter defibrillator, and 28 (14.0%) had undergone left cardiac sympathetic denervation. Most participants [*n* = 103 (51.8)] used beta-blocker and flecainide combination therapy, 85 (42.7%) used beta-blocker monotherapy, and 11 (5.5%) used flecainide monotherapy. After diagnosis, 34 (17.0%) had an arrhythmic event (defined as syncope, appropriate implantable cardioverter defibrillator shock, or sudden cardiac arrest), of whom 23 (11.5%) had a near-fatal arrhythmic event (defined as all of the above except for syncope). Characteristics of this population are presented in *Table 2*.

**Table 2:** Demographics and CPVT characteristics of participants using CPVT medication

	Adherent participants (*n* = 170)	Non-adherent participants (*n* = 30)	All participants using medication (*n* = 200)	*P*-value
Current age (median [IQR])	34.0 [22.0–52.0]	32.5 [22.8–42.0]	33.5 [22.0–50.2]	0.614
Gender, female (%)	98 (57.6)	24 (80.0)	122 (61.0)	0.035
Genotype (%)				0.787
Unknown	31 (18.2)	4 (13.3)	35 (17.5)	
Genotype-negative	9 (5.3)	2 (6.7)	11 (5.5)	
Genotype-positive	130 (76.5)	24 (80.0)	154 (77.0)	
*RYR2* variant carrier (%)	91 (53.5)	18 (60.0)	109 (54.5)	0.647
Age at diagnosis (median [IQR])	24.0 [12.0–40.0]	21.0 [15.2–35.8]	24.0 [13.0–40.0]	0.969
Autism (%)	11 (7.1)	3 (13.0)	14 (7.9)	0.566
Reason of presentation (%)				0.320
CPVT cascade screening	60 (35.3)	9 (30.0)	69 (34.5)	
Incidental finding	17 (10.0)	1 (3.3)	18 (9.0)	
Screening because of sudden cardiac death in family	13 (7.6)	1 (3.3)	14 (7.0)	
Cardiac symptoms	80 (47.1)	19 (63.3)	99 (49.5)	
Worst symptom prior to diagnosis (%)				0.203
Aborted cardiac arrest	27 (15.9)	7 (23.3)	34 (17.0)	
Asymptomatic	68 (40.0)	6 (20.0)	74 (37.0)	
Palpitations	19 (11.2)	5 (16.7)	24 (12.0)	
Syncope	56 (32.9)	12 (40.0)	68 (34.0)	
Symptoms after diagnosis^a^ (%)	30 (17.6)	4 (13.3)	34 (17.0)	0.752
Near-fatal symptoms after diagnosis^b^ (%)	22 (12.9)	1 (3.3)	23 (11.5)	0.226
Current oral medication (%)^c^				<0.001
Beta-blocker and flecainide	86 (50.9)	17 (56.7)	103 (51.8)	
Beta-blocker monotherapy	78 (46.2)	7 (23.3)	85 (42.7)	
Flecainide monotherapy	5 (3.0)	6 (20.0)	11 (5.5)	
Beta-blocker type; beta-1 selective (%)	53 (32.3)	5 (20.8)	58 (30.9)	0.368
Daily intake medicines more than once a day (%)	65 (38.2)	18 (60.0)	83 (41.5)	0.042
ICD implanted (%)	41 (24.1)	5 (16.7)	46 (23.0)	0.510
Left cardiac sympathetic denervation (%)	22 (12.9)	6 (20.0)	28 (14.0)	0.458
Follow-up frequency (%)				0.797
Never	5 (2.9)	2 (6.7)	7 (3.5)	
Once per 2 or 3 years	25 (14.7)	5 (16.7)	30 (15.0)	
Once a year	87 (51.2)	16 (53.3)	103 (51.5)	
More than once a year	40 (23.5)	5 (16.7)	45 (22.5)	
Other	13 (7.6)	2 (6.7)	15 (7.5)	

^a^Defined as syncope, aborted cardiac arrest, or appropriate implantable cardioverter defibrillator shock.

^b^Defined as aborted cardiac arrest or appropriate implantable cardioverter defibrillator shock.

^c^For one participant, the currently used CPVT medication was not known.

CPVT, catecholaminergic polymorphic ventricular tachycardia; ICD, implantable cardioverter defibrillator; IQR, interquartile range.

### Non-adherence to medication

The median total MARS-5 score of the participants was 24 [23–25]. Sixty-three (31.5%) participants had a total MARS-5 score of 25, reflecting perfect adherence. Thirty (15.0%) participants were defined as non-adherent to medical therapy. Only three (10.0%) non-adherent participants answered the unintentional non-adherence statement (‘I forget to take my medicine’) with ‘never’, while for the intentional adherence statements the percentage of non-adherent patients rating ‘never’ ranged from 40.0% to 63.3% (*Table 3*). In comparison, 74 (43.5%) adherent participants answered all the unintentional non-adherence statements with ‘never’. Only two (6.7%) non-adherent participants had a maximum score of 20 on the intentional non-adherence statements, meaning they had perfect intentional adherence and were defined as non-adherent based on their low score on the unintentional non-adherence statement. The median total score of the intentional adherence questions in the non-adherent patients was 17 [15–18].

**Table 3:** MARS-5 questionnaire: intentional and unintentional non-adherence

	Adherent participants (*n* = 170)	Non-adherent participants (*n* = 30)	All participants using CPVT medication (*n* = 200)
Median total MARS-5 score [IQR]	24 [24–25]	21 [19–22]	24 [23–25]
Unintentional adherence			
Median total score [IQR]	4 (4–5)	3 (3–4)	4 (4–5)
I forget to take my medicine (%)			
Very often	0 (0.0)	1 (3.3)	1 (0.5)
Often	0 (0.0)	4 (13.3)	4 (2.0)
Sometimes	15 (8.8)	14 (46.7)	29 (14.5)
Rarely	81 (47.6)	8 (26.7)	89 (44.5)
Never	74 (43.5)	3 (10.0)	77 (38.5)
Intentional adherence			
Median total score [IQR]	20 [20–20]	17 [15–18]	20 [20–20]
I alter the dose of my medicines (%)			
Very often	0 (0.0)	2 (6.7)	2 (1.0)
Sometimes	1 (0.6)	9 (30.0)	10 (5.0)
Rarely	14 (8.2)	7 (23.3)	21 (10.5)
Never	155 (91.2)	12 (40.0)	167 (83.5)
I stop taking my medicines for a while (%)			
Very often	0 (0.0)	2 (6.7)	2 (1.0)
Sometimes	1 (0.6)	5 (16.7)	6 (3.0)
Rarely	2 (1.2)	4 (13.3)	6 (3.0)
Never	167 (98.2)	19 (63.3)	186 (93.0)
I decide to miss out a dose (%)			
Very often	0 (0.0)	2 (6.7)	2 (1.0)
Often	0 (0.0)	1 (3.3)	1 (0.5)
Sometimes	0 (0.0)	3 (10.0)	3 (1.5)
Rarely	4 (2.4)	7 (23.3)	11 (5.5)
Never	166 (97.6)	17 (56.7)	183 (91.5)
I take less than instructed (%)			
Very often	0 (0.0)	3 (10.0)	3 (1.5)
Sometimes	0 (0.0)	7 (23.3)	7 (3.5)
Rarely	0 (0.0)	5 (16.7)	5 (2.5)
Never	170 (100.0)	15 (50.0)	185 (92.5)

This table shows the five 5-point Likert scale questions of the medication adherence rating scale (MARS-5) questionnaire. The first question reflects unintentional non-adherence. The subsequent questions reflect intentional non-adherence.

CPVT, catecholaminergic polymorphic ventricular tachycardia; IQR, interquartile range.

####  

##### Demographics and catecholaminergic polymorphic ventricular tachycardia characteristics

There were significantly more females in the non-adherent group compared to the adherent group [24 (80.0%) vs. 98 (57.6%), *P* = 0.035]. Furthermore, the prescribed medication differed between the two groups: 6 (20.0%) non-adherent patients used flecainide monotherapy vs. 5 (3.0%) adherent patients (*P* < 0.001), and significantly more non-adherent patients had to take their CPVT medicines more than once a day compared to adherent participants [18 (60.0%) vs. 65 (38.2%), *P* = 0.042]. The self-reported rate of symptoms after diagnosis was similar between non-adherent and adherent patients [4 (13.3%) vs. 30 (17.6%), *P* = 0.752; *Table 2*].

##### Beliefs about medication and illness perception

**Figure 2:**
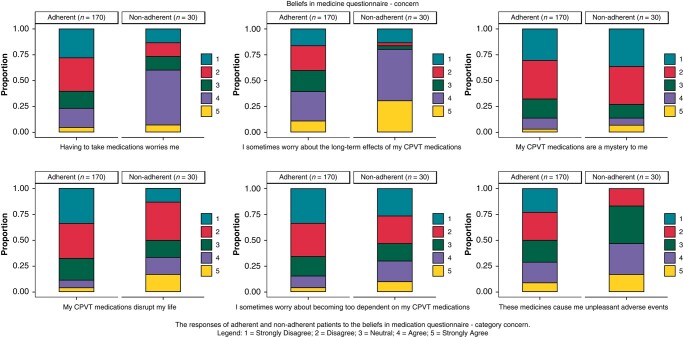
Agreement of adherent vs. non-adherent participants with statements regarding. The responses of adherent and non-adherent patients to the Beliefs about Medications Questionnaire, subcategory concern. Legend: 1 = strongly disagree; 2 = disagree; 3 = neutral; 4 = agree; 5 = strongly agree. The left columns represent the proportion of adherent patients (*n* = 170), and the right columns represent the non-adherent patients (*n* = 30).

Non-adherent patients had a significantly higher agreement with statements regarding concerns about CPVT medication compared to adherent patients [18.0 (15.2–21.8) vs. 14.0 (11.0–18.0), *P* < 0.001; *Table 4*]. Specifically, the agreement of non-adherent participants compared to adherent participants with the following statements was significantly different: ‘Having to take medications worries me’ (*P* < 0.001), ‘I sometimes worry about the long-term effects of my CPVT medications’ (*P* < 0.001), ‘My CPVT medications disrupt my life’ (*P* = 0.010), ‘These medicines cause me unpleasant adverse events’ (*P* = 0.010) (*Figure 2*). This was underlined by the answers of some participants to the optional free-text question ‘Do you have ideas on how the care for people with CPVT could be improved?’ at the end of the survey. One non-adherent participant mentioned: ‘I would like to know long-term effects of my beta-blocker, at the moment my cardiologist said to take for the rest of my life. How will that help me in the future and will there be adverse issues?’. Another non-adherent participant wrote: ‘I'd love to only have to take meds once a day but honestly if they had fewer side effects I'd take them however I needed to.’ A wide range of side effects, such as fatigue, nausea, mental problems including libido disorder, cold extremities, and dizziness, was reported by the participants. Fatigue was most commonly reported as a side effect by 76 (38.0%) participants.

**Table 4:** Beliefs about medication

	Adherent patients (*n* = 170)	Non-adherent patients (*n* = 30)	*P*-value
Concern about CPVT medication [IQR]	14.0 [11.0–18.0]	18.0 [15.2–21.8]	<0.001
Necessity CPVT medication [IQR]	18.0 [15.0–21.0]	19.0 [14.0–21.0]	0.806
Overuse medication in general [IQR]	8.0 [7.0–10.5]	8.5 [6.8–11.2]	0.724
Harm medication in general [IQR]	8.0 [6.0–10.0]	8.5 [6.8–11.0]	0.679

In this table, the median [IQR] of the total score of the beliefs about medication 5-point Likert questions are shown.

CPVT, catecholaminergic polymorphic ventricular tachycardia; IQR, interquartile range.

Non-adherent participants rated the statement ‘How much does CPVT affect you emotionally?’ of the illness perception questionnaire significantly higher compared to adherent participants [6.0 (4.0–8.0) vs. 5.0 (2.0–7.0), *P* = 0.032, *Table 5*]. One non-adherent participant mentioned in the final question regarding ideas on improvements in CPVT care: ‘It is a difficult condition, because I feel fine without meds and when I visit the cardiologist, I am reminded I could die and it takes some weeks to find my balance with this again.’ Another participant wrote: ‘We need help for the stress/anxiety as it can be overwhelming.’ Generally, a need for peer and psychological support was expressed by many participants.

**Table 5:** Illness perception questionnaire

	Adherent participants (*n* = 170)	Non-adherent participants (*n* = 30)	All participants using CPVT medication (*n* = 200)	*P*-value
How much does CPVT affect your life? [IQR]	6.0 [4.0–8.0]	8.0 [5.0–8.0]	6.0 [4.0–8.0]	0.102
How much control do you feel you have over CPVT? [IQR]	6.0 [4.0–8.0]	5.0 [3.2–8.0]	6.0 [4.0–8.0]	0.617
How much do you think your treatment can help CPVT? [IQR]	8.0 [7.0–10.0]	8.0 [5.0–10.0]	8.0 [7.0–10.0]	0.322
How much do you experience symptoms of CPVT? [IQR]	2.0 [1.0–5.0]	3.5 [2.0–5.8]	3.0 [1.0–5.0]	0.123
How concerned are you about CPVT? [IQR]	5.0 [3.0–8.0]	5.5 [4.0–8.0]	5.0 [3.0–8.0]	0.459
How well do you feel you understand your illness? [IQR]	8.0 [6.0–9.0]	8.0 [5.0–9.8]	8.0 [6.0–9.0]	0.689
How much does CPVT affect you emotionally? (e.g. does it make you angry, scared, upset, or depressed?) [IQR]	5.0 [2.0–7.0]	6.0 [4.0–8.0]	5.0 [2.0–7.0]	0.032

This table shows the median [IQR] of the self-reported agreement with above-mentioned statements on a scale from 0 to 10.

CPVT, catecholaminergic polymorphic ventricular tachycardia; IQR, interquartile range.

##### Independent associations with non-adherence

In multivariable analyses, female sex [odds ratio (OR) 3.7, 95% confidence interval (CI) 1.3–12.0, *P* = 0.019], flecainide monotherapy compared to flecainide and beta-blocker combination therapy (OR 6.8, 95%CI 1.6–31.0, *P* = 0.010), and a higher agreement with statements regarding concern about CPVT medication (OR 1.2, 95%CI 1.1–1.3, *P* < 0.001) were independently associated with a higher odds for non-adherence (*Table 6*).

**Table 6:** Multivariable logistic regression non-adherent vs. adherent participants

	OR	95% CI	*P*-value
Female sex	3.7	1.3–12.0	0.019
CPVT medicine (reference: beta-blocker and flecainide combination therapy)			
Beta-blocker monotherapy	0.6	0.2–1.5	0.266
Flecainide monotherapy	6.8	1.6–31.0	0.010
More than one daily intake of CPVT medicine	2.2	0.9–5.7	0.100
BMQ: concern	1.2	1.1–1.3	<0.001
BIPQ: emotionally affected	1.0	0.9–1.2	0.980

BIPQ, Brief Illness Perception Questionnaire; BMQ, Beliefs about Medications Questionnaire; CI, confidence interval; CPVT, catecholaminergic polymorphic ventricular tachycardia; OR, odds ratio.

### Adherence to lifestyle recommendations

The questions in the lifestyle section of the survey were not mandatory. Therefore, not all participants answered every question. Percentages are based on the total number of participants that provided an answer to the specific question.

**Figure 3:**
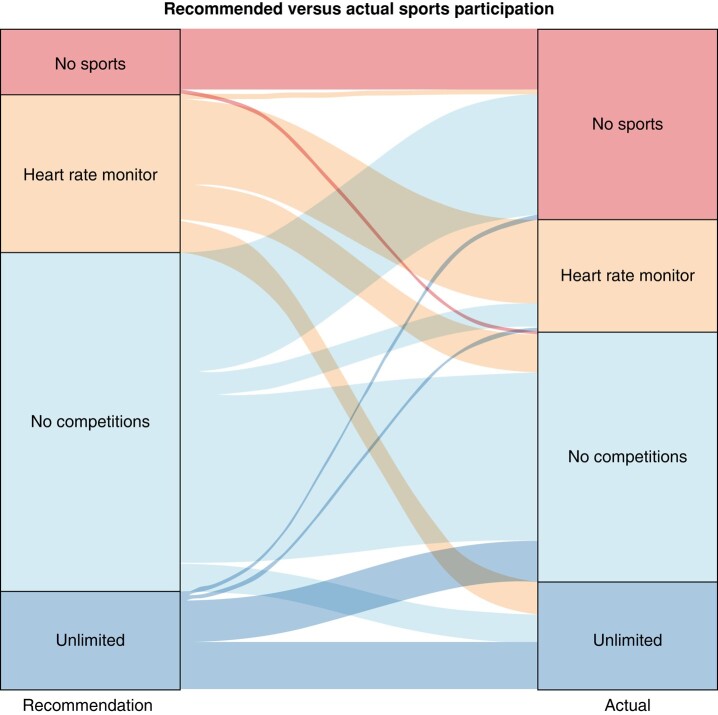
Physician recommended sports participation vs. actual sports participation. This figure shows the recommended (left) vs. actual (right) participation in sports of the survey participants. From top to bottom; no sports, meaning recommended/actually not involved in sports at all; heart rate monitor, meaning recommended/actually wearing a heart rate monitor; no competitions, meaning recommended/actually involved in sports, but not on a competitive level; unlimited, meaning recommended/actually involved in sports without any constraints. The vertical height of the categories and the thickness of the lines between the left and right panes represent the number of participants.

Most patients (*n* = 143/215 [66.8%]) had received lifestyle recommendations from their cardiologist, 63/215 (29.3%) patients did not receive lifestyle recommendations and 9/215 (4.1%) were unaware if recommendation had been given. Of the patients who received lifestyle recommendations, 14/142 (9.9%) participants were advised against practicing any sports, while 73/142 (51.4%) were allowed to practice non-competitive sports, and 34/142 (23.9%) were allowed to exercise using a heart rate monitor, while 21/142 (14.8%) were allowed to participate in sports activities without any constraints. Thirteen (10.7%) of the 121 participants who received a restrictive sports recommendation—i.e. either to use a heart rate monitor, to refrain from competitive sports, or to refrain from practicing any sports—were involved in sports without any limitation (*Figure 3*).

Prior to diagnosis, 152 patients had a median duration of sports performance of 6 [3–10] h/week. At present, 141 CPVT patients reported a median duration of sports performance of 4 [3–7] h/week. Most participants (*n* = 119/147, 81.0%) currently practiced sports on a recreational level, while 10 (6.8%) participants performed sports on a regional, national, or international level. Eighty-six of 209 (41.1%) participants noted being a little or very afraid of practicing sports. Furthermore, 112/207 (54.1%) participants wanted to be more involved in sports than they currently were, including 30 patients who indicated anxiety as one of the reasons that limited their sports participation.

## Discussion

This study is the first to assess adherence to medication and lifestyle recommendations in a large and representative international cohort of CPVT patients. Our results show that 15% of the participants were non-adherent to their prescribed medication. Female sex, flecainide monotherapy compared to flecainide, and beta-blocker combination therapy, and a high concern about CPVT medication were independently associated with non-adherence to medical therapy. Additionally, 11% of the participants who received a restrictive sports recommendation were involved in sports without any limitation. This knowledge could guide patient education aimed at therapy and lifestyle recommendations adherence and thereby hopefully also decrease arrhythmic events associated with non-adherence in patients with CPVT.

### Non-adherence measures in other populations

A previous study in patients with congenital long QT syndrome used pharmacy dispensing data from 68 patients and defined non-adherence as <80% of follow-up days with dispensed beta-blocker.^26^ With this method, 35 (51%) non-adherent patients were identified, which is significantly higher than we found in our study. Another more recent and large study regarding adherence to beta-blockers in 500 patients with congenital long QT syndrome used a similar approach of prescription data and nationwide registries and found that 38.4% of the patients were non-adherent, defined as patients with a treatment break of >60 days,^14^ Both studies presumably used a more objective method for measuring therapeutic non-adherence than in our study. The self-reported adherence questionnaire used in the current study reflects other aspects of adherence besides dispensing rates. Studies in other populations—elderly,^27^ patients using biologicals,^28^ and patients using statins chronically^21^—that also use MARS-5 to identify non-adherent patients, show more similar non-adherence rates (13%, 11%, and 7%, respectively). Indeed, in a large Swedish cohort of patients with a statin prescription after an ischaemic stroke, who had both filled in the MARS-5 questionnaire and whose dispensing rate was measured, the proportion of non-adherent patients was higher when using the dispensing rate method compared to the MARS-5 questionnaire (22% vs. 13%, respectively).^29^ In self-reported adherence questionnaires, participants might overestimate their adherence to the prescribed drug. Furthermore, it is conceivable that non-adherent patients were less motivated to participate in this survey. Conversely, picking up a prescription does not necessarily mean that a patient is actually taking the medication.

In clinical practice, self-reported adherence questionnaires in addition to dispensing rates and an unexpected increase in the presence and severity of ventricular arrhythmia on the exercise stress test^30–32^ might help a cardiologist to identify patients that are non-adherent to their CPVT medication.

### Intentional vs. unintentional non-adherence

The aforementioned different aspects of non-adherence might also be in part illustrated by the unintentional and intentional non-adherence questions within the MARS-5 itself. In our cohort, both adherent and non-adherent patients most frequently selected another option than ‘never’ to the unintentional non-adherence question ‘I forget to take my medicine’. This indicates that a significant number of patients in both groups tend to forget to take their medication occasionally.

With respect to intentional non-adherence, only two (6.7%) non-adherent participants responded with ‘never’ to every intentional non-adherent statement. Conversely, among the adherent participants, the majority (*n* = 149, 87.6%) rated all intentional non-adherence statements with ‘never’. This emphasizes the significant proportion of deliberate non-adherence within our cohort, highlighting the opportunity to reduce it.

### Factors associated with non-adherence

Female sex, flecainide monotherapy compared to beta-blocker and flecainide combination therapy and a higher agreement with statements related to concern about CPVT medication were independently associated with non-adherence. Alleviating concerns about CPVT medication might be of particular interest to reduce non-adherence. The association between concern about medication and intentional non-adherence has been previously reported in patients with inherited cardiac diseases^13^ and in other populations^33^ in combination with a low necessity belief about medication.

In our cohort, the higher concerns of CPVT medication in non-adherent participants was partially attributed to four statements. Importantly, non-adherent participants showed a greater level of agreement with statements related to having to take medication in general and apprehensions about the long-term effects of their medication. These apprehensions may contribute to the intentional non-adherence. This finding is particularly noteworthy because both beta-blockers and flecainide are essential and frequently used components of the cardiologist’s treatment arsenal. Despite common side effects, which were also observed by our study cohort, there is no evidence for long-term negative effects of these medications. Addressing the discrepancy between patients’ beliefs about their medicines and the evidence could potentially ease some of the concerns that lead to non-adherence to CPVT medication. In addition, open discussions between the cardiologist and the patient are essential to ensure that concerns from patients are being relayed to their physicians, and that recommendations given are understood.

The CIs of the ORs of flecainide monotherapy and female sex were very wide. This reflects either the small total number of non-adherent participants or a reduced robustness of the effect, and these associations should thus be regarded with caution. However, the knowledge of these associations might help clinicians to identify their non-adherent patients.

The majority of the non-adherent patients were in the reproductive age period (median age 32.5 [22.8–42.0] years). Therefore, one could speculate that women tend to be less adherent to their medication in the reproductive age, when concern about medication extends beyond themselves to their (potentially future) offspring. Non-adherence to medication during pregnancy is well-described in other conditions requiring chronic use of medication.^34^

Generally, flecainide monotherapy is prescribed in patients after they suffered from significant side effects on beta-blocker monotherapy or beta-blocker and flecainide combination therapy. Thus, the subgroup of patients on flecainide monotherapy is biased: some of these patients have probably already suffered from side effects, and, therefore, they might have bigger concerns about their medication compared to a previously treatment naïve CPVT population in whom CPVT medication is initiated, increasing the risk of non-adherence.

Furthermore, the high burden of side effects stresses the need for alternative therapeutic options for these patients. Catecholaminergic polymorphic ventricular tachycardia patients are generally young and active and frequent side effects such as fatigue or dizziness can have a huge impact on their life. Left cardiac sympathetic denervation is currently reserved for those patients who have had a syncope or documented ventricular tachycardia despite treatment with a beta-blocker and flecainide.^6^ Future studies should assess whether this procedure could be an effective treatment strategy in non-adherent patients who are less symptomatic and whether this could safely be accompanied by less medication or lower dosages. These studies should take into account the invasiveness of this procedure and the risk of long-term complications, such as left-sided dryness, unilateral facial flush, and contralateral hyperhidrosis.^35^ Yet, the majority of patients who had undergone a left cardiac sympathetic denervation reported they were satisfied with the procedure and would recommend it to others.^35^ Additionally, it is well-known that drug responses differ between individuals and are associated with variants in genes encoding enzymes involved in metabolizing drugs. Recently, it has been shown that a pharmacogenetic-guided drug prescription, taking these genetic factors into account, decreases the occurrence of side effects.^36^ This breakthrough might especially be useful for the CPVT patient population, in whom genetic testing for CPVT variants is already performed for regular care. Until then, improved patient education about CPVT medication is of the utmost importance.

Although it did not reach statistical significance in multivariable analyses, non-adherent participants were prescribed medication they had to take more than once daily significantly more often compared to adherent participants. Given the availability of long-acting formulations without disadvantages compared to more than once-daily prescriptions, these alternatives should be favoured within the CPVT population.

Lastly, the emotional impact of CPVT was not independently associated with non-adherence. Nevertheless, emotional and psychological care deserves the attention of the CPVT caretakers worldwide. In addition to a higher self-rated emotional impact of CPVT in the non-adherent participants, a substantial number of participants mentioned, without being specifically asked, that there currently is a lack of and a need for psychological care. In a survey that was excluded because it was filled in by the parent of a CPVT patient that was tragically deceased, the participant mentioned ‘My son’s CPVT was well-managed. He had returned to sport. He lived every day in fear of ‘dying again’ after cardiac arrest mid-swim race which led to his diagnosis. He took his own life … . Mental health support for patients is severely limited and lacking….’. This is also supported by a small cross-sectional study about psychosocial implications of living with CPVT, that underlined the importance of psychosocial support especially for young patients.^37^

### Lifestyle recommendations

We showed that the lifestyle recommendations received by the patients are very diverse and many patients (29%) did not receive—or could not recall receiving—lifestyle recommendations. Around half of the patients who did receive lifestyle recommendation were advised to avoid sports on a competitive level, in accordance with current European guidelines,^6^ while other guidelines are less stringent.^16^ Ideally, there should be an internationally uniform approach to lifestyle recommendations. However, this should not compromise an individualized and shared-decision making approach, as some patients may want to be more involved in sports than others, and in asymptomatic patients the CPVT phenotype might permit a more lenient approach. Also, it has been shown previously that the occurrence of events in well-informed and well-treated patients who remained competitive athletes was similar to non-athletes in a cohort of 63 CPVT patients.^15^ Indeed, some experts in the field advocate the continuation of competitive sports in some CPVT athletes when well-treated and well-informed.^16^ Additionally, experimental data in CPVT mice suggest that exercise training might actually reduce ventricular arrhythmia.^38^ The underlying mechanisms remain unclear. On the other hand, other stressful experiences, such as stimulating video games, may also trigger arrhythmic events in CPVT.^39,40^ Lastly, participating in sports is—understandably—accompanied by anxiety in many patients, and in our cohort 30 patients were less involved in sports that they would have liked, partly due to anxiety.

In summary, there is currently a lack of knowledge of lifestyle adaption in CPVT patients. Future studies are on the horizon that will shine more light on this topic, and will hopefully provide more evidence-based grounds for future guidelines and shared-decision making tools regarding lifestyle recommendations in CPVT.^41^

### Study limitations

Due to the study design, our results might have been biased due to non-responders. Non-adherent patients might generally be less likely to respond to a survey such as this one. We tried to make the risk as low possible by keeping the survey short, not stating the goal of the survey prior to entering, and by keeping it completely anonymous. Anonymity additionally reduces the risk of participants giving socially desirable answers and thereby acquiescence bias. Furthermore, MARS-5 is designed to reduce social desirability bias and sets a tone where non-adherence is considered normal.^18^ Lastly, due to the nature of the disease, non-adherent patients have a higher risk of symptoms and might therefore be deceased, leading to a survival bias.

## Conclusion and clinical implications

Non-adherence to medical therapy in CPVT patients is a significant challenge associated with higher concern regarding CPVT medications, including concerns about their long-term effects. Addressing these concerns might serve as a foundation to improve patient education aimed at therapy adherence. This should be a topic of open discussion during patient visits. In line with this, when initiating a treatment, a CPVT patient should be informed about the short- and long-term effects of their medication. Moreover, enhancing CPVT care could involve prescribing exclusively once-daily medication regimens, and integrating psychological support, and supportive discussions about optimizing safe sports participation. Future research should focus on establishing evidence-based lifestyle recommendations in CPVT.

## Supplementary Material

euae044_Supplementary_Data

## Data Availability

The data underlying this article will be shared on reasonable request to the corresponding author.

## References

[1] Leenhardt A , Lucet V, Denjoy I, Grau F, Ngoc DD, Coumel P. Catecholaminergic polymorphic ventricular tachycardia in children. A 7-year follow-up of 21 patients. Circulation 1995;91:1512–9. 7867192 10.1161/01.cir.91.5.1512

[2] van der Werf C , Kannankeril PJ, Sacher F, Krahn AD, Viskin S, Leenhardt A et al Flecainide therapy reduces exercise-induced ventricular arrhythmias in patients with catecholaminergic polymorphic ventricular tachycardia. J Am Coll Cardiol 2011;57:2244–54. 21616285 10.1016/j.jacc.2011.01.026 PMC3495585

[3] Hayashi M , Denjoy I, Extramiana F, Maltret A, Buisson NR, Lupoglazoff JM et al Incidence and risk factors of arrhythmic events in catecholaminergic polymorphic ventricular tachycardia. Circulation 2009;119:2426–34. 19398665 10.1161/CIRCULATIONAHA.108.829267

[4] Kannankeril PJ , Moore JP, Cerrone M, Priori SG, Kertesz NJ, Ro PS et al Efficacy of flecainide in the treatment of catecholaminergic polymorphic ventricular tachycardia: a randomized clinical trial. JAMA Cardiol 2017;2:759–66. 28492868 10.1001/jamacardio.2017.1320 PMC5548393

[5] Peltenburg PJ , Kallas D, Bos JM, Lieve KVV, Franciosi S, Roston TM et al An international multicenter cohort study on beta-blockers for the treatment of symptomatic children with catecholaminergic polymorphic ventricular tachycardia. Circulation 2022;145:333–44. 34874747 10.1161/CIRCULATIONAHA.121.056018

[6] Zeppenfeld K , Tfelt-Hansen J, de Riva M, Winkel BG, Behr ER, Blom NA et al 2022 ESC guidelines for the management of patients with ventricular arrhythmias and the prevention of sudden cardiac death. Eur Heart J 2022;43:3997–4126. 36017572 10.1093/eurheartj/ehac262

[7] van der Werf C , Zwinderman AH, Wilde AA. Therapeutic approach for patients with catecholaminergic polymorphic ventricular tachycardia: state of the art and future developments. Europace 2012;14:175–83. 21893508 10.1093/europace/eur277

[8] Roston TM , Vinocur JM, Maginot KR, Mohammed S, Salerno JC, Etheridge SP et al Catecholaminergic polymorphic ventricular tachycardia in children: analysis of therapeutic strategies and outcomes from an international multicenter registry. Circ Arrhythm Electrophysiol 2015;8:633–42. 25713214 10.1161/CIRCEP.114.002217 PMC4472494

[9] van der Werf C , Wilde AA. Catecholaminergic polymorphic ventricular tachycardia: important messages from case reports. Europace 2011;13:11–3. 20851823 10.1093/europace/euq330

[10] Roston TM , Yuchi Z, Kannankeril PJ, Hathaway J, Vinocur JM, Etheridge SP et al The clinical and genetic spectrum of catecholaminergic polymorphic ventricular tachycardia: findings from an international multicentre registry. Europace 2018;20:541–7. 28158428 10.1093/europace/euw389 PMC6059141

[11] Celiker A , Erdogan I, Karagoz T, Ozer S. Clinical experiences of patients with catecholaminergic polymorphic ventricular tachycardia. Cardiol Young 2009;19:45–52. 10.1017/S1047951108003338 19102802

[12] Loar RW , Bos JM, Cannon BC, Ackerman MJ. Sudden cardiac arrest during sex in patients with either catecholaminergic polymorphic ventricular tachycardia or long-QT syndrome: a rare but shocking experience. J Cardiovasc Electrophysiol 2015;26:300–4. 25514987 10.1111/jce.12600

[13] O'Donovan CE , Waddell-Smith KE, Skinner JR, Broadbent E. Predictors of beta-blocker adherence in cardiac inherited disease. Open Heart 2018;5:e000877. 30613409 10.1136/openhrt-2018-000877 PMC6307606

[14] Kroll J , Butt JH, Jensen HK, Fosbol EL, Camilla HBJ, Winkel BG et al Beta-blocker adherence among patients with congenital long QT syndrome: a nationwide study. Eur Heart J Qual Care Clin Outcomes 2022;9:76–84. 35438152 10.1093/ehjqcco/qcac017

[15] Ostby SA , Bos JM, Owen HJ, Wackel PL, Cannon BC, Ackerman MJ. Competitive sports participation in patients with catecholaminergic polymorphic ventricular tachycardia: a single center's early experience. JACC Clin Electrophysiol 2016;2:253–62. 29766881 10.1016/j.jacep.2016.01.020

[16] Ackerman MJ , Zipes DP, Kovacs RJ, Maron BJ. Eligibility and disqualification recommendations for competitive athletes with cardiovascular abnormalities: task force 10: the cardiac channelopathies: a scientific statement from the American Heart Association and American College of Cardiology. J Am Coll Cardiol 2015;66:2424–8. 26542662 10.1016/j.jacc.2015.09.042

[17] Castor EDC . (2019). Castor electronic data capture [online] https://castoredc.com. [Internet].

[18] Chan AHY , Horne R, Hankins M, Chisari C. The medication adherence report scale: a measurement tool for eliciting patients’ reports of nonadherence. Br J Clin Pharmacol 2020;86:1281–8. 31823381 10.1111/bcp.14193 PMC7319010

[19] Garcia-Marcos PW , Brand PL, Kaptein AA, Klok T. Is the MARS questionnaire a reliable measure of medication adherence in childhood asthma? J Asthma 2016;53:1085–9. 27177241 10.1080/02770903.2016.1180699

[20] Almardini R , Taybeh EO, Alsous MM, Hawwa AF, McKeever K, Horne R et al A multiple methods approach to determine adherence with prescribed mycophenolate in children with kidney transplant. Br J Clin Pharmacol 2019;85:1434–42. 30845359 10.1111/bcp.13911 PMC6595357

[21] Ladova K , Matoulkova P, Zadak Z, Macek K, Vyroubal P, Vlcek J et al Self-reported adherence by MARS-CZ reflects LDL cholesterol goal achievement among statin users: validation study in the Czech Republic. J Eval Clin Pract 2014;20:671–7. 24917035 10.1111/jep.12201

[22] de Ridder D , Theunissen N. De rol van ziektepercepties in therapietrouw bij hypertensie. Gedrag en Gezondheid 2003;31:237–45.

[23] Horne R , Weinman J, Hankins M. The beliefs about medicines questionnaire: the development and evaluation of a new method for assessing the cognitive representation of medication. Psychol Health 1999;14:1–24.

[24] Broadbent E , Petrie KJ, Main J, Weinman J. The brief illness perception questionnaire. J Psychosom Res 2006;60:631–7. 16731240 10.1016/j.jpsychores.2005.10.020

[25] de Raaij EJ , Schroder C, Maissan FJ, Pool JJ, Wittink H. Cross-cultural adaptation and measurement properties of the Brief Illness Perception Questionnaire-Dutch Language Version. Man Ther 2012;17:330–5. 22483222 10.1016/j.math.2012.03.001

[26] Waddell-Smith KE , Li J, Smith W, Crawford J, Skinner JR, Cardiac Inherited Disease Group New Zealand. Beta-blocker adherence in familial long QT syndrome. Circ Arrhythm Electrophysiol 2016;9:e003591. 27516460 10.1161/CIRCEP.115.003591

[27] Irshaidat S , Gustafsson M, Norberg H. Self-reported medication adherence among older people admitted to hospital: a descriptive study. Drugs Real World Outcomes 2023;10:23–9. 36703097 10.1007/s40801-023-00352-8 PMC9944347

[28] van der Groef R , de Jong PHP, Hijnen DJ, van der Woude CJ, van Laar JAM, van der Kuy PHM et al Impact of the first SARS-CoV-2 lockdown on adherence to biological treatment in patients with immune-mediated inflammatory diseases in the Netherlands. Patient Prefer Adherence 2023;17:167–74. 36698858 10.2147/PPA.S392290 PMC9869789

[29] Norberg H , Sjolander M, Glader EL, Gustafsson M. Self-reported medication adherence and pharmacy refill adherence among persons with ischemic stroke: a cross-sectional study. Eur J Clin Pharmacol 2022;78:869–77. 35156130 10.1007/s00228-022-03284-4 PMC9005421

[30] Haugaa KH , Leren IS, Berge KE, Bathen J, Loennechen JP, Anfinsen OG et al High prevalence of exercise-induced arrhythmias in catecholaminergic polymorphic ventricular tachycardia mutation-positive family members diagnosed by cascade genetic screening. Europace 2010;12:417–23. 20106799 10.1093/europace/eup448

[31] Peltenburg PJ , Pultoo SNJ, Tobert KE, Bos JM, Lieve KVV, Tanck M et al Repeatability of ventricular arrhythmia characteristics on the exercise-stress test in RYR2-mediated catecholaminergic polymorphic ventricular tachycardia. Europace 2023;25:619–26. 36369981 10.1093/europace/euac177 PMC9934990

[32] Crotti L , Brugada P, Calkins H, Chevalier P, Conte G, Finocchiaro G et al From gene-discovery to gene-tailored clinical management: 25 years of research in channelopathies and cardiomyopathies. Europace 2023;25:euad180. 37622577 10.1093/europace/euad180 PMC10450790

[33] Clifford S , Barber N, Horne R. Understanding different beliefs held by adherers, unintentional nonadherers, and intentional nonadherers: application of the necessity-concerns framework. J Psychosom Res 2008;64:41–6. 18157998 10.1016/j.jpsychores.2007.05.004

[34] Matsui D . Adherence with drug therapy in pregnancy. Obstet Gynecol Int 2012;2012:796590. 22242026 10.1155/2012/796590 PMC3253470

[35] Waddell-Smith KE , Ertresvaag KN, Li J, Chaudhuri K, Crawford JR, Hamill JK et al Physical and psychological consequences of left cardiac sympathetic denervation in long-QT syndrome and catecholaminergic polymorphic ventricular tachycardia. Circ Arrhythm Electrophysiol 2015;8:1151–8. 26224781 10.1161/CIRCEP.115.003159

[36] Swen JJ , van der Wouden CH, Manson LE, Abdullah-Koolmees H, Blagec K, Blagus T et al A 12-gene pharmacogenetic panel to prevent adverse drug reactions: an open-label, multicentre, controlled, cluster-randomised crossover implementation study. Lancet 2023;401:347–56. 36739136 10.1016/S0140-6736(22)01841-4

[37] Richardson E , Spinks C, Davis A, Turner C, Atherton J, McGaughran J et al Psychosocial implications of living with catecholaminergic polymorphic ventricular tachycardia in adulthood. J Genet Couns 2018;27:549–57. 28940060 10.1007/s10897-017-0152-1

[38] Faggioni M , Hwang HS, van der Werf C, Nederend I, Kannankeril PJ, Wilde AA et al Accelerated sinus rhythm prevents catecholaminergic polymorphic ventricular tachycardia in mice and in patients. Circ Res 2013;112:689–97. 23295832 10.1161/CIRCRESAHA.111.300076 PMC3601570

[39] Lawley CM , Tester M, Sanatani S, Prendiville T, Beach CM, Vinocur JM et al Life-threatening cardiac arrhythmia and sudden death during electronic gaming: an international case series and systematic review. Heart Rhythm 2022;19:1826–33. 37850595 10.1016/j.hrthm.2022.08.003

[40] Lawley CM , Skinner JR, Turner C. Syncope due to ventricular arrhythmia triggered by electronic gaming. N Engl J Med 2019;381:1180–1. 31532968 10.1056/NEJMc1905537

[41] Moulson N , Petek BJ, Ackerman MJ, Churchill TW, Day SM, Kim JH et al Rationale and design of the ORCCA (Outcomes Registry for Cardiac Conditions in Athletes) Study. J Am Heart Assoc 2023;12:e029052. 37259981 10.1161/JAHA.122.029052 PMC10382007

