# Supplementary material for: Insights into adherence to medication and lifestyle recommendations in an international cohort of patients with catecholaminergic polymorphic ventricular tachycardia
Source: Europace. 2024 Feb 13;26(2):euae044. doi: 10.1093/europace/euae044 (PMC10886442; doi:10.1093/europace/euae044)
Supplement: euae044_Supplementary_Data [file euae044_supplementary_data.pdf]

Supplementary Material

Supplementary Methods

General CPVT related questions

The following information was collected:

- Demographic characteristics: current age, sex, language, home country, way of recruitment, level of education, behavior problem such as autism or attention deficit hyperactivity disorder.
- CPVT diagnosis: age at diagnosis, reason of first presentation cardiologist, (age at) symptoms (separately assessed syncope, resuscitation, palpitations) prior to diagnosis, family history of CPVT and CPVT-related symptoms, genotype (if genotype positive: *RYR2* or *CASQ2* or other gene variant), most recent exercise test
- CPVT therapy and follow-up: beta-blocker, type of beta-blocker, flecainide, other medication, number of daily dosages, side-effects, in case of no medication: reason, other CPVT therapies: implantable cardioverter defibrillator implantation, left cardiac sympathetic denervation, (age at) symptoms (separately assessed syncope, resuscitation, palpitations, ICD shock, inappropriate ICD shock), frequency of cardiologic follow-up.

Lifestyle

The lifestyle survey contained the following questions:

1. Did your (pediatric) cardiologist recommend a certain lifestyle with regard to performing sports/physical exercise?
2. How would you describe the lifestyle recommendation in your own words?
3. What description fits the lifestyle recommendation from your treating physician best?
   a. I am not allowed to do sports
   b. I can do sports, but not on a competitive level
   c. I can do sports, but I have to use a heart rate monitor
   d. I can do sports without any constraints
4. What description fits your actual engagement in sport/physical exercise best?
   a. I don't do sports at all
   b. I do sports, but not on a competitive level
   c. I do sports, but use a heart rate monitor

    d. I do sports without any constraints
5. What type of sport do you perform? Please select all applicable options.
6. How many hours in total per week do you perform sport/physical exercise?
7. On what level do you perform sport?
    a. recreational
    b. recreational competition
    c. regional competition
    d. national competition
    e. international competition
8. Were you involved in sport/physical exercise prior to the CPVT diagnosis?
9. What type of sport(s) were your involved in prior to the CPVT diagnosis? Please select all applicable options.
10. How many hours in total per week did you perform sport/physical exercise prior to the CPVT diagnosis?
11. On what level did you perform sports prior to the CPVT diagnosis? Please select the highest level you ever performed.
12. Do you want to be involved in more sports/physical exercise than you currently are?
13. Why are you currently less involved in sports/physical exercise than you would like?
    a. because it was recommended by my (pediatric) cardiologist
    b. I am scared
    c. I have too little time
    d. I don't feel like it
    e. other
14. Are you afraid of performing sports/physical exercise because you have CPVT?
    a. yes, very much
    b. yes, a little
    c. sometimes
    d. no, not really
    e. no, not at all

Social media recruitment

The recruitment of CPVT patients was limited to CPVT specific social media platforms, channeling only patients with CPVT. The survey was posted on a CPVT patients Facebook group on December 27, 2022 and reposted on March 7, 2023 and again on June 9, 2023. Furthermore, the survey was shared in mailings and posted on the website and social media accounts of the following patient organizations: the Sudden Arrhythmic Deaths Syndrome (SADS) foundation, SADS UK, and the Cardiac Risk in the Young (CRY).

Sample size calculation

The sample size calculation was based on a previous study using similar methods (855 participants in an online survey study after 1 years of enrollment, including patients with a Fontan procedure with an estimated prevalence of 4-8 in 10.000 (1), compared to an estimated CPVT prevalence of 1 in 10.000 (2)). In an adherence survey study in patients with inherited cardiac disease, in which 618 individuals were sent the invitation to the survey directly the response rate was 34% after three months (3). In a pilot survey study in the Dutch cohort of patients with CPVT, 38.3% of the patients recruited at a patient information day completed an online survey similar to the current study. At the time of writing the protocol, 711 (53.1%) patients are from a Dutch or English or French speaking country in the International CPVT Registry (4). To be able to describe a representative sample, we aimed to include a total number of 30% (n=213) CPVT patients.

Supplemental references

1. Schumacher KR, Stringer KA, Donohue JE, Yu S, Shaver A, Caruthers RL, et al. Social media methods for studying rare diseases. Pediatrics. 2014;133(5):e1345-53.

2. Zeppenfeld K, Tfelt-Hansen J, de Riva M, Winkel BG, Behr ER, Blom NA, et al. 2022 ESC Guidelines for the management of patients with ventricular arrhythmias and the prevention of sudden cardiac death. Eur Heart J. 2022;43(40):3997-4126.

3. O'Donovan CE, Waddell-Smith KE, Skinner JR, Broadbent E. Predictors of beta-blocker adherence in cardiac inherited disease. Open Heart. 2018;5(2):e000877.

4. Peltenburg PJ, Kallas D, Bos JM, Lieve KVV, Franciosi S, Roston TM, et al. An International Multicenter Cohort Study on beta-Blockers for the Treatment of Symptomatic Children With Catecholaminergic Polymorphic Ventricular Tachycardia. Circulation. 2022;145(5):333-44.
